# Relationship of Anxiety, Depression, Stress, and Post-Traumatic Stress Disorder Symptoms with Disease Severity in Acutely Ill Hospitalized COVID-19 Patients

**DOI:** 10.3390/bs13090734

**Published:** 2023-09-01

**Authors:** Dijana Lucijanic, Alma Mihaljevic Peles, Nevenka Piskac Zivkovic, Marko Lucijanic, Matija Kranjcevic, Lana Muzinic Marinic

**Affiliations:** 1 Department of Psychiatry, Referral Centre for Stress-Related Disorders of the Ministry of Health, Centre University Hospital Dubrava, Avenija Gojka Šuška 6, 10000 Zagreb, Croatia; 2 Clinical Hospital Centre Zagreb, Psychiatric Clinic, Kispaticeva 12, 10000 Zagreb, Croatia; 3 Department for Chemistry and Biochemistry, School of Medicine, Salata 3, 10000 Zagreb, Croatia; 4 Special Hospital Radiochirurgia, Ulica dr. Franje Tuđmana 4, 10431 Sveta Nedjelja, Croatia; 5 Hematology Department, University Hospital Dubrava, Av. Gojka Suska 6, 10000 Zagreb, Croatia; 6 School of Medicine, University of Zagreb, Salata 3, 10000 Zagreb, Croatia; 7 University of Applied Health Sciences in Zagreb, Mlinarska Street 38, 10000 Zagreb, Croatia

**Keywords:** anxiety, COVID-19, depression, PTSD, stress

## Abstract

We aimed to investigate depression, anxiety, stress, and PTSD symptoms and their relationship with disease severity in acutely ill hospitalized Coronavirus disease 2019 (COVID-19) patients. A single-center cross-sectional observational survey study screening for psychiatric symptoms using the Depression, Anxiety and Stress Scale—21 Items (DASS-21) and the Impact of Events Scale-Revised (IES-R) questionnaires was performed including a total of 169 acutely ill COVID-19 patients. All patients were adults and of white race and developed respiratory insufficiency during hospitalization. Demographic, clinical and laboratory data were evaluated as predictors of psychiatric symptoms. We hypothesized that higher intensity of COVID-19 symptoms and higher oxygen requirement would be associated with occurrence of depression, anxiety, stress, and PTSD symptoms. Depressive symptoms were absent in 29%, mild in 16%, moderate in 27.8%, severe in 10.7% and extremely severe in 16.6% patients. Anxiety symptoms were absent in 43.8%, mild in 6.5%, moderate in 17.2%, severe in 5.3% and extremely severe in 27.2% patients. Stress symptoms were absent in 78.7%, mild in 4.7%, moderate in 7.1%, severe in 7.7%, and extremely severe in 1.8% patients. A total of 60.9% patients had no PTSD symptoms, 16% had undiagnosed symptoms, and 23.1% met the criteria for a PTSD diagnosis. All psychiatric symptoms were more pronounced in female patients, depression and anxiety symptoms were associated with prior chronic obstructive pulmonary disease. Only depressive symptoms were significantly associated with higher intensity of COVID-19 symptoms and higher oxygen requirement. Acutely ill hospitalized COVID-19 patients presented a high prevalence of emergent psychiatric sequelae, especially in females, and more severe COVID-19 influenced mostly the severity of depressive symptoms.

## 1. Introduction

The cCoronavirus disease 2019 (COVID-19) pandemic substantially challenged stress resilience and mental health outcomes, especially among vulnerable populations such as patients with previous psychiatric disorders, severely ill COVID-19 patients, health care workers, children, and elderly [1]. Mental health consequences may occur in weeks, months or longer after the infection. Hospitalized COVID-19 patients suffering from more severe forms of COVID-19 may be particularly susceptible to adverse mental health conditions [2].

COVID-19 is a multisystemic disease affecting dominantly respiratory system. Although a majority of severe acute respiratory syndrome coronavirus 2 (SARS-CoV-2) infected patients experience no or only mild symptoms, prior to wide-spread availability of vaccination, a substantial proportion of patients (15–20%) developed acute respiratory insufficiency, requiring oxygen supplementation and thus needed to be hospitalized [3]. Subsequent vaccination program and occurrence of novel, less aggressive viral strains significantly reduced the number of patients presenting with severe and critical forms of the disease. Despite benefits of vaccination, even among those with breakthrough infections [4], vaccine hesitancy, inadequate immunization among elderly and immunosuppressed patients, as well as waning effects of vaccine-induced immunization remain substantial problems for global control of the COVID-19 pandemic [5,6].

Several putative mechanisms by which COVID-19 may induce psychological symptoms have been proposed [7,8]. Clinical, post-mortem, animal, in vitro, and cell culture studies demonstrated that coronaviruses are potentially neurotropic and can induce neuronal injuries [9]. Notwithstanding possible brain infiltration, “cytokines storm” involved in the immune response to coronaviruses may cause psychiatric symptoms by precipitating neuroinflammation [10].

Also, the brain is highly sensitive to the change in the arterial concentration of oxygen [11]; it ineluctably sustains the hypoxic stress. Decreased oxygen content might lead to neuronal injury in the brains of chronic obstructive pulmonary disease (COPD) patients, which is demonstrated by clinical symptoms such as mood disorders and neuropsychological deficits. Furthermore, the systemic inflammation is accompanied by a hypoxic injury that may exacerbate the neuronal injury. A large number of patients with intermittent hypoxia manifest a series of symptoms related to the injury to the nervous system, which is exhibited as a deficiency in memory, learning, and decision-making ability. In addition, the hypoxic damage in the brains of other patients is manifested as depression, anxiety, physical disabilities, and neuropsychological deficits [12]. Some studies demonstrate that hypoxia is associated with the changes in the brain structure, including volume atrophy and a decrease in the gray matter in the amygdala, hippocampus, anterior cingulate cortex, prefrontal cortex, and other regions [13].

Being hospitalized for a serious illness can negatively affect mental health and which may be further intensified when hospitalized with COVID-19 due to the exceptional circumstances within and outside the hospital during the pandemic [14]. Patients hospitalized with COVID-19 are isolated to avoid the virus spreading to other patients or health care staff. COVID-19 patients are thus limited in their access to social support, and their only in-person contact is with health care staff in full personal protective equipment. Studies have highlighted that isolation itself may have psychological implications [15]. Finally, the host immune response to SARS-CoV-2 infection and the persistent psychological stress before and during infection, as well as adverse effects of treatment, such as insomnia caused by corticosteroids, have also been suggested as possible mechanisms [16].

A number of studies have investigated psychiatric symptoms among COVID-19 patients and health care workers. Depression, anxiety, and stress symptoms seem to be highly prevalent among hospitalized COVID-19 patients [17,18,19], similar to previous severe acute respiratory syndrome (SARS) outbreaks [20]. Specific approaches like cognitive behavioral therapy might help in reducing the burden COVID-19 imposes on mental health of affected patients [19]. Pandemic also negatively affects psychological distress of community dwellers [21,22] and may increase rates of acute psychiatric admissions due to acute psychosis [23]. It also negatively affects mental health of the health care workers [24,25].

To better understand clinical context in which psychiatric symptoms may occur in COVID-19 patients and considering the lack of published data on depression, anxiety, stress, and PTSD symptoms in the acute setting, we decided to prospectively investigate this issue in a real-life cohort of acutely ill hospitalized COVID-19 patients with severe or critical clinical presentation. We hypothesized that higher intensity of COVID-19 symptoms and higher oxygen requirement would be associated with occurrence of depression, anxiety, stress, and PTSD symptoms.

## 2. Materials and Methods

### 2.1. Participants and Their Medical Evaluation

We performed a cross-sectional evaluation of 169 hospitalized COVID-19 patients hospitalized in the University hospital Dubrava, Zagreb, Croatia in period from November 2020 to May 2021. All patients were tested positive for COVID-19 by polymerase chain reaction or antigen test and had compatible clinical symptoms. All patients were adults and of white race. Patients were treated according to the contemporary guidelines, receiving oxygen supplementation, dexamethasone, and low molecular weight heparins in addition to their chronic and other acute therapies [26,27]. Demographic, clinical, and laboratory data were recorded at the time of hospital admission and were obtained through analysis of written and electronical medical records. Severity of COVID-19 at admission was classified using the World health organization recommendations into mild, moderate, severe, and critical [26]. Modified early warning score (MEWS) was used to assess intensity of COVID-19 symptoms [28]. Patient comorbidities were recorded as individual diseases and as a cumulative comorbidity burden quantified using the Charlson comorbidity index [29]. Functional status of patients at admission was classified using the Eastern cooperative oncology group (ECOG) scale [30]. Patients with disorders of consciousness, poor physical condition, patients currently requiring mechanical ventilation or in the intensive care unit, and who have severe communication difficulties (dementia, deafness, intellectual difficulties) were excluded from the study. All included patients developed severe or critical form of disease (respiratory insufficiency) and required oxygen supplementation. Patients with critical intensity of symptoms were allowed to participate upon stabilization of their clinical condition and transferred to medical wards.

### 2.2. Evalution of Psychiatric Symptoms

Written informed consent was obtained from all participants prior to entry into the study. After the introductory interview and informed consent, demographic data was obtained, and then, psychological questionnaires were administered. Study questionnaires included general questionnaire on demographic variables, psychiatric scales the Depression, Anxiety and Stress Scale—21 Items (DASS-21) Croatian adaptation [31], and the Impact of Events Scale-Revised (IES-R) [11].

DASS-21 is a short form of the DASS-42, a self-reported scale designed to measure negative emotional states of depression, anxiety, and stress. It has 21 items with seven questions for each condition, depression, anxiety, and stress, and each item having four possible answers scored from 0 to 3 points. The score for each item is multiplied by factor two to comply with original scale comprising 42 items. For depression, scores 10–13, 14–20, 21–27, and ≥28 points are considered to represent mild, moderate, severe, and extremely severe symptoms, respectively. For anxiety, scores 8–9, 10–14, 15–19, and ≥20 points are considered to represent mild, moderate, severe, and extremely severe symptoms, respectively. For stress, scores 15–18, 19–25, 26–33, and ≥34 points are considered to represent mild, moderate, severe, and extremely severe symptoms, respectively. The scale was shown to have good internal consistency and concurrent validity [32,33].

IES-R is a short self-reported scale designed to measure PTSD. It has 22 items, each scored from 0 to 4 points. The total IES-R score was divided into 0–23, 24–32, and ≥33 points considered to represent absence of PTSD symptoms, undiagnosed symptoms, and meeting the criteria for PTSD diagnosis, respectively. The scale was shown to have good internal consistency and concurrent validity [34,35].

Total DASS-21 score (Cronbach’s alpha 0.930), DASS-21 scores for individual scales (Cronbach’s alpha values of 0.788, 0.878, and 0.837 for depression, anxiety, and stress, respectively) and IES-R score (Cronbach’s alpha 0.918) showed good to excellent internal consistency in our sample as well, implying the reliability of scales.

The median duration of the disease until filling out of the questionnaire was 15 days. The questionnaires were administered to patients with investigators using personal protective equipment.

### 2.3. Statistical Methods

The normality of the distribution of numerical variables was tested with the Kolmogorov–Smirnov test. Numerical variables were non-normally distributed and presented as median and interquartile range (IQR) and were compared between groups using the Mann–Whitney U test and the Kruskal–Wallis ANOVA test. Spearman correlation was used to compare two numerical variables. Categorical variables were compared using the chi square test. Multivariate analyses were performed using the logistic regression models, with presence of individual psychiatric symptoms being dependent variables for each model, and intensity of COVID-19 symptoms and required oxygen supplementation, age, sex, Charlson comorbidity index, and prior psychiatric treatment being independent variables. *p*-values < 0.05 were considered statistically significant. All analyses were performed using the MedCalc statistical software version 22.007 (MedCalc Software Ltd., Ostend, Belgium).

### 2.4. Ethical Considerations

The study was conducted in accordance with the Declaration of Helsinki and approved by the Institutional Review Board of the University hospital Dubrava (Nm. 2020/1012-05).

### 2.5. Patients’ Characteristics

A total of 169 patients were analyzed. There were 105 (62.1%) male and 64 (37.9%) female patients. Median age was 65 years, IQR (57–71). Median Charlson comorbidity index was 3 points, IQR (2–4). At the time of hospital admission 134 (79.3%) patients had severe and 17 (10.1%) patients had critical severity of COVID-19 symptoms. Median MEWS score was 3 points, IQR (1–4). Median C reactive protein (CRP) was 72.75 mg/L, IQR (31.4–141.4).

## 3. Results

### 3.1. Depression

The median score for depressive symptoms was 14 points, IQR (8–22). Depressive symptoms were absent in 49 (29%), mild in 27 (16%), moderate in 47 (27.8%), severe in 18 (10.7%), and extremely severe in 28 (16.6%) patients. Relationship of presence of depressive symptoms with clinical characteristics are shown in Table 1.

**Figure 1:**
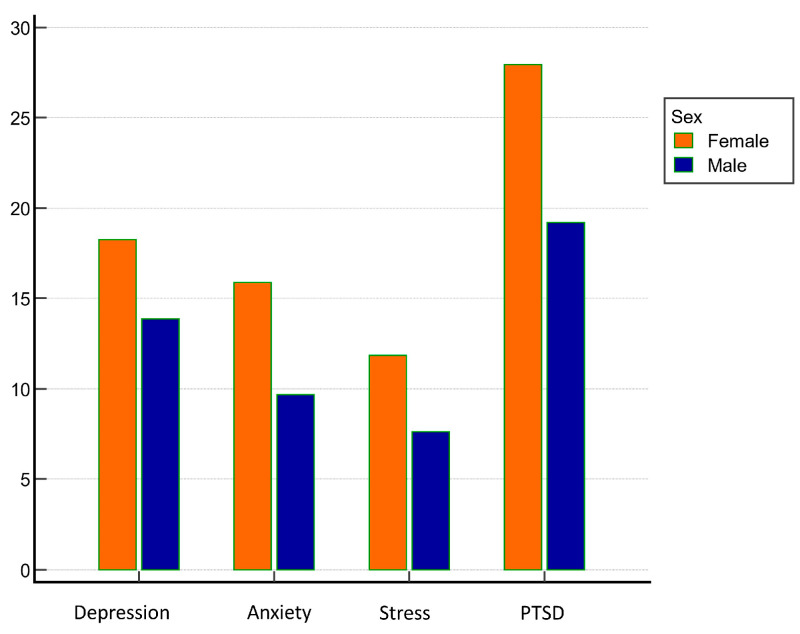
Sex differences in assessed psychiatric symptoms’ measurements.

Patients with the presence of depressive symptoms at a statistically significant level were more often of female sex (Figure 1), had chronic obstructive pulmonary disease, had a more pronounced intensity of symptoms of COVID-19 at the time of hospital admission, required oxygen supplementation at higher flows, less frequently had deep vein thrombosis, and had lower red blood cell distribution width (RDW), lower D-dimers, and lower procalcitonin. They also more frequently felt that their lives were in greater danger due to COVID-19 (*p* < 0.05 for all analyses). Neither age, CRP, nor other investigated parameters were significantly associated with the presence or severity of depressive symptoms during hospitalization.

### 3.2. Anxiety

The median score for anxiety symptoms was 8 points, IQR (2–20). Anxiety symptoms were absent in 74 (43.8%), mild in 11 (6.5%), moderate in 29 (17.2%), severe in 9 (5.3%), and extremely severe in 46 (27.2%) patients. Relationship of presence of anxiety symptoms with clinical characteristics are shown in Table 2.

Patients with the presence of anxiety symptoms at a statistically significant level were more often female (Figure 1), felt more threatened by COVID-19, more often had COPD, more often had bacteremia during the stay, and lower D-dimers on admission (*p* < 0.05 for all analyses). There was no statistically significant association of the presence or severity of anxiety symptoms during hospitalization with the severity of COVID-19 at admission, with CRP or with other examined characteristics.

### 3.3. Stress

The median score for stress symptoms was 6 points, IQR (2–12.5). Stress symptoms were absent in 133 (78.7%), mild in 8 (4.7%), moderate in 12 (7.1%), severe in 13 (7.7%), and extremely severe in 3 (1.8%) patients. Relationship of presence of stress symptoms with clinical characteristics are shown in Table 3.

Patients with the presence stress symptoms at a statistically significant level were more often female (Figure 1), felt more threatened by COVID-19, were more likely to have been previously psychiatrically treated, and had lower D-dimer values and lower procalcitonin values at admission (*p* < 0.05 for all analyzes).

### 3.4. PTSD

The median IES-R score was 20 points, IQR (11–31.25). A total of 103 (60.9%) patients had no PTSD symptoms, 27 (16%) had undiagnosed symptoms, and 39 (23.1%) met the criteria for a PTSD diagnosis. Relationship of presence of PTSD symptoms with clinical characteristics are shown in Table 4.

Patients with PTSD symptoms present at a statistically significant level were more often female (Figure 1), felt that their lives were more at risk due to COVID-19, had lower values of RDW, procalcitonin, and creatinine during hospital admission, and were previously psychiatrically treated (*p* < 0.05 for all analyses).

### 3.5. Multivariate Analyses

We performed a series of multivariate logistic regression models investigating relationship of presence of individual psychiatric symptoms with severity of COVID-19 symptoms, required intensity of oxygen supplementation, age, sex, Charlson comorbidity index, and previous psychiatric treatment. Results are presented in Table 5. Presence of depressive symptoms was significantly associated with higher intensity of COVID-19 symptoms and was nearly significant for female sex, whereas presence of anxiety, stress, and PTSD symptoms remained significantly associated with female sex in the context of aforementioned clinically relevant adjustments.

## 4. Discussion

We performed a cross-sectional study evaluating prevalence of depression, anxiety, stress, and PTSD symptoms among acutely ill, hospitalized COVID-19 patients, using the personal protective equipment as patients were considered infectious and required mandatory isolation. As we report, psychopathology is prevalent in this patient population and demographic and clinical characteristics affect mostly the severity of depressive symptoms. Depressive symptoms were the only symptoms significantly associated with the intensity of COVID-19 symptoms. Also, female patients might require special consideration since all investigated psychiatric symptoms were more prevalent among female than male patients, even after adjustments for clinically relevant variables.

In order of prevalence, depressive symptoms were the most frequent psychiatric disorder affecting 71% of patients, followed by anxiety symptoms affecting 56.2% patients, and PTSD symptoms with 23.1% patients meeting the criteria for its diagnosis, and additional 16% having undiagnosed symptoms, followed by stress symptoms present in 21.3% patients. One should bear in mind that we investigated a cohort of real-life patients with high prevalence of severe or critical COVID-19 symptoms upon their clinical stabilization. Our results are not readily comparable or translatable to other clinical settings, as other studies evaluated more heterogenous patient population with milder forms of COVID-19 in different stages of development.

Despite male patients experiencing higher severity of COVID-19, especially among elderly [36], females might be more vulnerable regarding mental health as shown in the current, as well as in multiple previous studies [37,38,39]. This is most likely due to a number of biological, psychological, and cultural factors associated with female sex [39]. Females generally tend to assume a caregiving role in their families, which is very demanding to balance with work and household tasks, making them vulnerable and predisposing them to mental health issues in situations of overload [40].

Similar to acute setting, in the post-illness stage, depressed mood, insomnia, anxiety, irritability, memory impairment, fatigue, and traumatic memories were frequently reported in the literature as well [41]. The social situation to which COVID-19 survivors return is completely different from that of SARS and Middle East respiratory syndrome (MERS) (diseases caused by coronaviruses) survivors. These differences are relevant for the prevalence of psychiatric disorders in both acute and post-illness stages. In the COVID-19 era, unlike the previous SARS and MERS outbreaks, fear for shortage of medical facilities such as ventilators can further increase stress. Staying at the intensive care unit (ICU) is a risk factor for developing psychiatric disorders by itself. In 2018, a large study among almost 5000 ICU survivors showed that prevalence of post-traumatic stress disorder was 46%, that of anxiety was 40%, and that of depression was 22% [42].

Depression among hospitalized patients is often unrecognized, undiagnosed, and therefore untreated. Feasibility of screening for depression during hospitalization, or whether depression is associated with poorer outcomes, longer hospital stays, and higher readmission rates are still insufficiently explored. The prevalence of depression ranged from 5% to 60%, with a median of 33%, among hospitalized patients prior to COVID-19 [43].

The association between COPD and psychiatric disorders, in particular generalized anxiety, panic disorder, and depression, has been acknowledged for many years. As the findings of the current study show, having prior COPD was associated mostly with presence of anxiety, whereas no significant association was determined regarding other psychiatric symptoms. In recent years, the role of the immune system in the pathogenesis of chronic disease has been extensively studied. This trend is also prominent in psychiatry and systemic inflammation as well as localized modulation of microglial cell activity in the central nervous system (CNS) have been associated with most psychiatric conditions. Depression in particular may be associated with systemic inflammation and higher serum concentrations of inflammatory biomarkers such as interleukin (IL)-1 and tumor necrosis factor-alpha (TNF-a), though the nature of the association has yet to be clearly elucidated. A recent study [44] found elevated levels of IL-2, IL-6, and interferon-gamma (IFN-γ) in patients suffering from co-morbid COPD and depression compared to healthy controls, though the implications of these findings remain uncertain. On the opposite, results obtained from the cohort of the ECLIPSE study [45] indicate that there is no significant association between inflammatory biomarkers and symptoms of depression in individuals suffering from COPD. Similarly, we could not identify higher inflammatory burden measured through laboratory biomarkers of inflammation to be associated with presence of particular psychiatric symptoms.

Patients with the presence of depressive symptoms during hospitalization in our study, at a statistically significant level, had a more pronounced intensity of symptoms of COVID-19 at the time of hospital admission and required oxygen supplementation at higher flows. Hypoxia in the brain leads to oxidative stress, alterations of white matter, and alterations of endothelial cells, which can ultimately result in permanent cognitive defects and dementia. It modifies neuronal functions by altering the synthesis of neurotransmitters. High levels of carbon dioxide may activate the respiratory center of the brain stem, triggering anxiety in attempt to alert the body of possible suffocation. Interestingly, the onset of depression has also been demonstrated in COPD patients experiencing hypoxemia. In addition, hypoxemia can enhance systemic inflammation, and it has been demonstrated to induce nuclear factor κB, the master regulator of cellular inflammatory responses resulting in systemic inflammation [46]. Andelid and colleagues found hypoxemia after COPD exacerbations to be associated with systemic neutrophilic activity [47]. Moreover, it is hypothesized that hypoxia of adipose tissue in obese COPD patients might be an important source of systemic inflammation [48]. Hypoxemia-associated oxidative stress is a potential organic mechanism for the onset of depressive symptoms [49]. Supporting this, Forlenza and colleagues found increased serum levels of a biomarker for oxidative damage, 8-hydroxy-2′-deoxyguanosine, in depressed patients [50]. Translating this phenomenon to COPD, increased levels of oxidative stress have also been found in COPD patients [51]. Oxidative stress regulates inflammation in COPD, and decreased levels of the antioxidant glutathione are found after severe and very severe exacerbations (89.2% and 52.3%, respectively) relative to stable COPD. Taken together, hypoxemia and oxidative stress, directly and/or indirectly through associated systemic inflammation, might be involved in the onset of brain-associated comorbidities in COPD patients.

CNS viral infections [52], inflammatory process, and cerebral hypoxia [53] have substantial impact on cognitive functions producing transient or permanent cognitive impairment. Limbic and associated brain structures such as the hippocampus and basal ganglia contain more enzymes that are involved in inflammatory responses than other areas. Therefore, there is an increased risk of developing deficits in neurocognitive processes like memory, attention, and emotion [54]. Research demonstrates that poorer cognitive function is associated with increased risk of depression, social withdrawal, and dependence [55] and can contribute to decreased quality of life.

Main limitations of our study are being a single center study and limited number of included patients, thus reflecting the statistical power of some of the presented analyses. Our findings are representative of a high volume tertiary referral center for treatment of most severe COVID-19 patients. The study was performed during the alpha SARS-CoV-2 viral strain dominated wave of the disease. Thus we were unable to estimate how potential vaccination could affect our measurements, as well as how occurrence and clinical correlations of investigated psychiatric symptoms might be related to infection by other viral strains. Inclusion of patients of only white race may also be considered as a limitation regarding generalizability of our findings. Due to cross-sectional study design, no causal relationship between investigated variables can be inferred.

## 5. Conclusions

Acutely ill hospitalized COVID-19 patients presented a high prevalence of emergent psychiatric sequelae, and clinical and demographic characteristics influenced mostly the severity of depressive symptoms. Female patients might require special considerations since all investigated psychiatric symptoms were more prevalent among female than male patients, even after adjustments for clinically relevant variables. Higher than average incidence of major depression, anxiety, and stress, all high-burden non-communicable conditions associated with years of life lived with disability, is expected in survivors. These findings motivate further follow-up studies of mental health among patients recovering from COVID-19 and other serious infections and impose the need for heightened clinical surveillance of those recovering from a severe illness.

## Figures and Tables

**Table 1:** Relationship of presence of depressive symptoms with clinical characteristics.

	No Depressive Symptoms (N = 49)	Depressive Symptoms (N = 120)	*p*-Value
Age (years), median and IQR	65 (59–71)	64.5 (57–72)	0.906
Sex			0.214 +
Male	34/49 (69.4%)	71/120 (59.2%)	
Female	15/49 (30.6%)	49/120 (40.8%)	
Level of education			0.672
Ground school	5/49 (10.2%)	18/120 (15%)	
High school	30/49 (61.2%)	60/120 (50%)	
Bachelor	7/49 (14.3%)	15/120 (12.5%)	
University	6/49 (12.2%)	22/120 (18.3%)	
Postgraduate doctoral degree	1/49 (2%)	4/120 (3.3%)	
Working status			0.394
Employed	13/49 (26.5%)	45/120 (37.5%)	
Unemployed	2/49 (4.1%)	4/120 (3.3%)	
Pension	34/49 (69.4%)	71/120 (59.2%)	
Partnership status			0.998
Marriage	34/49 (69.4%)	84/120 (70%)	
Extramarital union	1/49 (2%)	3/120 (2.5%)	
In a relationship	1/49 (2%)	2/120 (1.7%)	
Divorced	3/49 (6.1%)	6/120 (5%)	
A widower	8/49 (16.3%)	21/120 (17.5%)	
Not in a relationship	2/49 (4.1%)	4/120 (3.3%)	
Feeling their life is endangered due to COVID-19			0.001 * +
Not at all	19/49 (38.8%)	22/120 (18.3%)	
Little	9/49 (18.4%)	25/120 (20.8%)	
Moderately	17/49 (34.7%)	29/120 (24.2%)	
Seriously	3/49 (6.1%)	31/120 (25.8%)	
Very strong	1/49 (2%)	13/120 (10.8%)	
Charlson comorbidity index median and IQR	3 (2–4)	3 (2–4)	0.639
Prevously psychiatrically treated	6/49 (12.2%)	15/120 (12.5%)	0.964
Arterial hypertension	31/49 (63.3%)	70/120 (58.3%)	0.553
Diabetes	12/49 (24.5%)	30/120 (25%)	0.944
Hyperlipidemia	11/49 (22.4%)	19/120 (15.8%)	0.307
Adipositas	16/49 (32.7%)	45/120 (37.5%)	0.552
Smoking	2/49 (4.1%)	9/120 (7.5%)	0.513
Alcohol use	3/49 (6.1%)	4/120 (3.3%)	0.415
COPD	2/49 (4.1%)	10/120 (8.3%)	0.512 +
Asthma	1/49 (2%)	7/120 (5.8%)	0.440
Heart failure	8/49 (16.3%)	16/120 (13.3%)	0.613
Atrial fibrillation	5/49 (10.2%)	10/120 (8.3%)	0.767
Coronary disease	7/49 (14.3%)	16/120 (13.3%)	0.870
Periferial arterial disease	1/49 (2%)	4/120 (3.3%)	1.000
Cronical renal disease	7/49 (14.3%)	8/120 (6.7%)	0.138
Cronical hepatal disease	1/49 (2%)	2/120 (1.7%)	1.000
Active malignant disease	5/49 (10.2%)	12/120 (10%)	1.000
Transplanted organ	3/49 (6.1%)	4/120 (3.3%)	0.415
Rheumatic disease	2/49 (4.1%)	7/120 (5.8%)	1.000
WHO severity of COVID-19 at admission			0.452
Mild	4/49 (8.2%)	9/120 (7.5%)	
Moderate	3/49 (6.1%)	2/120 (1.7%)	
Heavy	38/49 (77.6%)	96/120 (80%)	
Critical	4/49 (8.2%)	13/120 (10.8%)	
MEWS symptom intensity, median and IQR	2 (1–3)	3 (2–4)	<0.001 * +
Pneumonia	45/49 (91.8%)	107/120 (89.2%)	0.780
Duration of disease symptoms, median and IQR	15 (11–20)	15 (11–21)	0.972
ECOG functional status, median and IQR	2 (1–2)	2 (1–3)	0.128
Peak required oxygen flow during hospitalization, median and IQR	6 (3–14)	8 (4–15)	0.397 +
Duration of hospitalization, median and IQR	14 (9–19)	11 (8–19)	0.213
Treatment in the intensive care unit	6/49 (12.2%)	19/120 (15.8%)	0.551
Treatment with mechanical ventilation	2/49 (4.1%)	7/120 (5.8%)	1.000
Bacteremia	1/49 (2%)	11/120 (9.2%)	0.183
Arterial thrombosis	1/49 (2%)	2/120 (1.7%)	1.000
Venous thrombosis	10/49 (20.4%)	10/120 (8.3%)	0.027 *
Heavy bleeding	0/49 (0%)	2/120 (1.7%)	1.000
Death during hospitalization	2/49 (4.1%)	7/120 (5.8%)	1.000
Leukocytes (×10^9^/L), median and IQR	7.6 (6.13–10.55)	8.5 (5.6–12.1)	0.894
Absolute number of neutrophil granulocytes (×10^9^/L), median and IQR	6.6 (4.36–9.2)	6.8 (4.2–10.1)	0.892
Absolute number of lymphocytes (×10^9^/L), median and IQR	0.9 (0.6–1.38)	0.8 (0.55–1.14)	0.511
Hemoglobin (g/L), median and IQR	136 (125–146.5)	133 (119.75–145)	0.546
RDW (%), median and IQR	14 (13.3–15.6)	13.8 (13.2–14.73)	0.368 +
Platelets (×10^9^/L), median and IQR	227.5 (166.25–308)	243 (170–324)	0.697
NLR, median and IQR	8.6 (3.77–12.56)	7.8 (4.31–12.63)	0.647
SII, median and IQR	1697.3 (595.16–4268.84)	1750.9 (948.71–3511.75)	0.857
CRP (mg/L), median and IQR	74.7 (27.53–135.9)	72 (35.38–139.93)	0.882
IL6 (pg/mL), median and IQR	24.4 (18.11–27.37)	16.3 (5.73–42.72)	0.705
Ferritin (µg/L), median and IQR	843 (409–1242)	767 (342.5–1527.5)	0.885
D-dimers (mg/L FEU), median and IQR	1.3 (0.8–4.15)	0.9 (0.57–2.2)	0.073 +
Procalcitonin (ng/mL), median and IQR	0.2 (0.08–0.27)	0.2 (0.07–0.22)	0.346 +
Creatinine (mmol/L), median and IQR	80 (66.5–98.5)	78 (67–97.5)	0.878

* statistically significant at level *p* < 0.05 when the Depression, Anxiety and Stress Scale—21 Items (DASS-21) score is stratified according to the absence or presence of symptoms. + statistically significant at level *p* < 0.05 when DASS-21 score is used as a continuous variable. Abbreviations: IQR—interquartile range; COVID-19—Coronavirus disease 2019; COPD—chronic obstructive pulmonary disease; WHO—the World health organization; MEWS—modified early warning score; ECOG—Eastern cooperative oncology group scale; RDW—red blood cell distribution width; NLR—neutrophil to lymphocyte ratio; SII—systemic inflammatory index derived from NLR multiplied by platelet count; CRP—C reactive protein; IL—interleukin; FEU—fibrin equivalent units.

**Table 2:** Relationship of presence of anxiety symptoms with clinical characteristics.

	No Anxiety Symptoms (N = 74)	Anxiety Symptoms (N = 95)	*p*-Value
Age (years), median and IQR	65.5 (55.25–70.75)	65 (59–72.5)	0.443
Sex			0.025 * +
Male	53/74 (71.6%)	52/95 (54.7%)	
Female	21/74 (28.4%)	43/95 (45.3%)	
Level of education			0.142
Ground school	7/74 (9.5%)	16/95 (16.8%)	
High school	38/74 (51.4%)	52/95 (54.7%)	
Bachelor	8/74 (10.8%)	14/95 (14.7%)	
University	16/74 (21.6%)	12/95 (12.6%)	
Postgraduate doctoral degree	4/74 (5.4%)	1/95 (1.1%)	
Working status			0.372
Employed	27/74 (36.5%)	31/95 (32.6%)	
Unemployed	1/74 (1.4%)	5/95 (5.3%)	
Pension	46/74 (62.2%)	59/95 (62.1%)	
Partnership status			0.733
Marriage			
Extramarital union	52/74 (70.3%)	66/95 (69.5%)	
In a relationship	2/74 (2.7%)	2/95 (2.1%)	
Divorced	2/74 (2.7%)	1/95 (1.1%)	
A widower	3/74 (4.1%)	6/95 (6.3%)	
Not in a relationship	11/74 (14.9%)	18/95 (18.9%)	
Feeling their life is endangered due to COVID-19			0.001 * +
Not at all	28/74 (37.8%)	13/95 (13.7%)	
Little	15/74 (20.3%)	19/95 (20%)	
Moderately	19/74 (25.7%)	27/95 (28.4%)	
Seriously	7/74 (9.5%)	27/95 (28.4%)	
Very strong	5/74 (6.8%)	9/95 (9.5%)	
Charlson comorbidity index, median and IQR	3 (2–4)	3 (2–4)	0.457
Prevously psychiatrically treated	6/74 (8.1%)	15/95 (15.8%)	0.133
Arterial hypertension	42/74 (56.8%)	59/95 (62.1%)	0.482
Diabetes	16/74 (21.6%)	26/95 (27.4%)	0.391
Hyperlipidemia	16/74 (21.6%)	14/95 (14.7%)	0.245
Adipositas	27/74 (36.5%)	34/95 (35.8%)	0.925
Smoking	5/74 (6.8%)	6/95 (6.3%)	1.000
Alcohol use	4/74 (5.4%)	3/95 (3.2%)	0.700
COPD	1/74 (1.4%)	11/95 (11.6%)	0.010 *
Asthma	2/74 (2.7%)	6/95 (6.3%)	0.468
Heart failure	9/74 (12.2%)	15/95 (15.8%)	0.503
Atrial fibrillation	7/74 (9.5%)	8/95 (8.4%)	0.814
Coronary disease	9/74 (12.2%)	14/95 (14.7%)	0.628
Periferial arterial disease	2/74 (2.7%)	3/95 (3.2%)	1.000
Cronical renal disease	8/74 (10.8%)	7/95 (7.4%)	0.435
Cronical hepatal disease	1/74 (1.4%)	2/95 (2.1%)	1.000
Active malignant disease	6/74 (8.1%)	11/95 (11.6%)	0.457
Transplanted organ	2/74 (2.7%)	5/95 (5.3%)	0.469
Rheumatic disease	3/74 (4.1%)	6/95 (6.3%)	0.733
WHO severity of COVID-19 at admission			0.609
Mild	5/74 (6.8%)	8/95 (8.4%)	
Moderate	2/74 (2.7%)	3/95 (3.2%)	
Heavy	57/74 (77%)	77/95 (81.1%)	
Critical	10/74 (13.5%)	7/95 (7.4%)	
MEWS symptom intensity, median and IQR	2 (1–4)	3 (1.5–4)	0.755
Pneumonia	67/74 (90.5%)	85/95 (89.5%)	0.819
Duration of disease symptoms, median and IQR	16 (12–21)	13 (10.5–18.5)	0.114
ECOG functional status, median and IQR	2 (1–3)	2 (1–3)	0.792
Peak required oxygen flow during hospitalization, median and IQR	7.5 (4–15)	6 (4–15)	0.593
Duration of hospitalization, median and IQR	12.5 (8.25–17.75)	11 (8.5–19.5)	0.790
Treatment in the intensive care unit	10/74 (13.5%)	15/95 (15.8%)	0.679
Treatment with mechanical ventilation	3/74 (4.1%)	6/95 (6.3%)	0.733
Bacteremia	2/74 (2.7%)	10/95 (10.5%)	0.049 *
Arterial thrombosis	0/74 (0%)	3/95 (3.2%)	0.257
Venous thrombosis	9/74 (12.2%)	11/95 (11.6%)	0.907
Heavy bleeding	0/74 (0%)	2/95 (2.1%)	0.505
Death during hospitalization	2/74 (2.7%)	7/95 (7.4%)	0.302
Leukocytes (×10^9^/L), median and IQR	7.4 (5.95–11.55)	8.5 (5.38–11.7)	0.753
Absolute number of neutrophil granulocytes (×10^9^/L), median and IQR	6.5 (4.55–9.94)	6.9 (4.1–9.72)	0.834
Absolute number of lymphocytes (×10^9^/L), median and IQR	0.9 (0.6–1.35)	0.8 (0.55–1.2)	0.343
Hemoglobin (g/L), median and IQR	135 (122.25–142)	134 (120–147.75)	0.594
RDW (%), median and IQR	13.8 (13.3–14.65)	13.8 (13.2–14.98)	0.944
Platelets (×10^9^/L), median and IQR	237 (171.25–309.5)	243.5 (164.5–322.5)	0.996
NLR, median and IQR	7.4 (4.52–13.01)	8.2 (3.93–12.45)	0.734
SII, median and IQR	1732.8 (812.77–3821.14)	1750.9 (897.4–3756.1)	0.919
CRP (mg/L), median and IQR	86.2 (42.58–146.43)	68.3 (30.48–136.98)	0.252
IL6 (pg/mL), median and IQR	14.5 (7.29–26.63)	24.2 (5.36–55.98)	0.934
Ferritin (µg/L), median and IQR	850 (475–1587.5)	693.5 (323.75–1503.75)	0.462
D-dimers (mg/L FEU), median and IQR	1.3 (0.66–4.08)	0.9 (0.57–1.71)	0.040 * +
Procalcitonin (ng/mL), median and IQR	0.2 (0.09–0.26)	0.2 (0.07–0.22)	0.153
Creatinine (mmol/L), median and IQR	82 (66.25–98.75)	77 (67–96)	0.478

* statistically significant at level *p* < 0.05 when the Depression, Anxiety and Stress Scale—21 Items (DASS-21) score is stratified according to the absence or presence of symptoms. + statistically significant at level *p* < 0.05 when DASS-21 score is used as a continuous variable. Abbreviations: IQR—interquartile range; COVID-19—Coronavirus disease 2019; COPD—chronic obstructive pulmonary disease; WHO—the World health organization; MEWS—modified early warning score; ECOG—Eastern cooperative oncology group scale; RDW—red blood cell distribution width; NLR—neutrophil to lymphocyte ratio; SII—systemic inflammatory index derived from NLR multiplied by platelet count; CRP—C reactive protein; IL—interleukin; FEU—fibrin equivalent units.

**Table 3:** Relationship of presence of stress symptoms with clinical characteristics.

	No Stress Symptoms (N = 133)	Stress Symptoms (N = 36)	*p*-Value
Age (years), median and IQR	65 (58–72)	63 (56.75–68.5)	0.311
Sex			0.004 * +
Male	90/133 (67.7%)	15/36 (41.7%)	
Female	43/133 (32.3%)	21/36 (58.3%)	
Level of education			0.277
Ground school	16/133 (12%)	7/36 (19.4%)	
High school	68/133 (51.1%)	22/36 (61.1%)	
Bachelor	20/133 (15%)	2/36 (5.6%)	
University	23/133 (17.3%)	5/36 (13.9%)	
Postgraduate doctoral degree	5/133 (3.8%)	0/36 (0%)	
Working status			0.716
Employed	45/133 (33.8%)	13/36 (36.1%)	
Unemployed	4/133 (3%)	2/36 (5.6%)	
Pension	84/133 (63.2%)	21/36 (58.3%)	
Partnership status			0.893
Marriage	91/133 (68.4%)	27/36 (75%)	
Extramarital union	3/133 (2.3%)	1/36 (2.8%)	
In a relationship	3/133 (2.3%)	0/36 (0%)	
Divorced	8/133 (6%)	1/36 (2.8%)	
A widower	23/133 (17.3%)	6/36 (16.7%)	
Not in a relationship	5/133 (3.8%)	1/36 (2.8%)	
Feeling their life is endangered due to COVID-19			0.011 *
Not at all	39/133 (29.3%)	2/36 (5.6%)	
Little	31/133 (23.3%)	3/36 (8.3%)	
Moderately	32/133 (24.1%)	14/36 (38.9%)	
Seriously	32/133 (24.1%)	14/36 (38.9%)	
Very strong	8/133 (6%)	6/36 (16.7%)	
Charlson comorbidity index, median and IQR	3 (2–4)	3 (1–4)	0.254
Prevously psychiatrically treated	13/133 (9.8%)	8/36 (22.2%)	0.045 * +
Arterial hypertension	82/133 (61.7%)	19/36 (52.8%)	0.335
Diabetes	35/133 (26.3%)	7/36 (19.4%)	0.397
Hyperlipidemia	27/133 (20.3%)	3/36 (8.3%)	0.096
Adipositas	50/133 (37.6%)	11/36 (30.6%)	0.435
Smoking	8/133 (6%)	3/36 (8.3%)	0.703
Alcohol use	7/133 (5.3%)	0/36 (0%)	0.348
COPD	7/133 (5.3%)	5/36 (13.9%)	0.134
Asthma	6/133 (4.5%)	2/36 (5.6%)	0.679
Heart failure	19/133 (14.3%)	5/36 (13.9%)	0.952
Atrial fibrillation	13/133 (9.8%)	2/36 (5.6%)	0.741
Coronary disease	20/133 (15%)	3/36 (8.3%)	0.415
Periferial arterial disease	5/133 (3.8%)	0/36 (0%)	0.586
Cronical renal disease	13/133 (9.8%)	2/36 (5.6%)	0.741
Cronical hepatal disease	2/133 (1.5%)	1/36 (2.8%)	0.515
Active malignant disease	13/133 (9.8%)	4/36 (11.1%)	0.761
Transplanted organ	5/133 (3.8%)	2/36 (5.6%)	0.642
Rheumatic disease	5/133 (3.8%)	4/36 (11.1%)	0.098
WHO severity of COVID-19 at admission			0.125
Mild	11/133 (8.3%)	2/36 (5.6%)	
Moderate	2/133 (1.5%)	3/36 (8.3%)	
Heavy	108/133 (81.2%)	26/36 (72.2%)	
Critical	12/133 (9%)	5/36 (13.9%)	
MEWS symptom intensity, median and IQR	3 (1–4)	3 (1–3)	0.934
Pneumonia	118/133 (88.7%)	34/36 (94.4%)	0.531
Duration of disease symptoms, median and IQR	15 (11–19)	15 (11.75–23.25)	0.271
ECOG functional status, median and IQR	2 (1–3)	2 (1–3)	0.564
Peak required oxygen flow during hospitalization, median and IQR	6 (4–15)	9.5 (4–16)	0.574
Duration of hospitalization, median and IQR	12 (8–19)	12 (9.75–21.25)	0.674
Treatment in the intensive care unit	17/133 (12.8%)	8/36 (22.2%)	0.157
Treatment with mechanical ventilation	6/133 (4.5%)	3/36 (8.3%)	0.403
Bacteremia	7/133 (5.3%)	5/36 (13.9%)	0.134
Arterial thrombosis	2/133 (1.5%)	1/36 (2.8%)	0.515
Venous thrombosis	15/133 (11.3%)	5/36 (13.9%)	0.771
Heavy bleeding	2/133 (1.5%)	0/36 (0%)	1.000
Death during hospitalization	6/133 (4.5%)	3/36 (8.3%)	0.403
Leukocytes (×10^9^/L), median and IQR	7.9 (5.78–11.18)	8.5 (4.95–12.65)	0.830
Absolute number of neutrophil granulocytes (×10^9^/L), median and IQR	6.6 (4.34–9.55)	7 (4.33–11.33)	0.608
Absolute number of lymphocytes (×10^9^/L), median and IQR	0.8 (0.55–1.32)	0.9 (0.62–1.06)	0.825
Hemoglobin (g/L), median and IQR	135 (118.5–145)	133.5 (125.75–143)	0.945
RDW (%), median and IQR	13.9 (13.28–15.05)	13.6 (13.2–14.03)	0.055
Platelets (×10^9^/L), median and IQR	239.5 (170–311.75)	241 (150–337.5)	0.742
NLR, median and IQR	7.8 (4.33–13.01)	8 (3.97–11.95)	0.887
SII, median and IQR	1726.8 (821.81–3865.65)	1886.8 (945.02–3324.39)	0.823
CRP (mg/L), median and IQR	74.1 (34.5–132.93)	64.1 (30.48–149.58)	0.776
IL6 (pg/mL), median and IQR	24.4 (8.27–28.05)	7 (5.36–50.95)	0.509
Ferritin (µg/L), median and IQR	761.5 (373.25–1358)	940 (373.5–1572)	0.656
D-dimers (mg/L FEU), median and IQR	1.1 (0.62–3.35)	0.8 (0.57–1.72)	0.147
Procalcitonin (ng/mL), median and IQR	0.2 (0.08–0.25)	0.1 (0.06–0.16)	0.021 * +
Creatinine (mmol/L), median and IQR	81 (67.5–101.5)	74 (56.75–83.25)	0.032 *

* statistically significant at level *p* < 0.05 when the Depression, Anxiety and Stress Scale—21 Items (DASS-21) score is stratified according to the absence or presence of symptoms. + statistically significant at level *p* < 0.05 when DASS-21 score is used as a continuous variable. Abbreviations: IQR—interquartile range; COVID-19—Coronavirus disease 2019; COPD—chronic obstructive pulmonary disease; WHO—the World health organization; MEWS—modified early warning score; ECOG—Eastern cooperative oncology group scale; RDW—red blood cell distribution width; NLR—neutrophil to lymphocyte ratio; CRP—C reactive protein; SII—systemic inflammatory index derived from NLR multiplied by platelet count; IL—interleukin; FEU—fibrin equivalent units.

**Table 4:** Relationship of presence of PTSD symptoms with clinical characteristics.

	No PTSD Symptoms (N = 103)	PTSD Symptoms (N = 66)	*p*-Value
Age (years), median and IQR	65 (56.5–71)	64.5 (59–72.5)	0.591
Sex			<0.001 * +
Male	76/103 (73.8%)	29/66 (43.9%)	
Female	27/103 (26.2%)	37/66 (56.1%)	
Level of education			0.188
Ground school	9/103 (8.7%)	14/66 (21.2%)	
High school	57/103 (55.3%)	33/66 (50%)	
Bachelor	15/103 (14.6%)	7/66 (10.6%)	
University	17/103 (16.5%)	11/66 (16.7%)	
Postgraduate doctoral degree	4/103 (3.9%)	1/66 (1.5%)	
Working status			0.846
Employed	36/103 (35%)	22/66 (33.3%)	
Unemployed	3/103 (2.9%)	3/66 (4.5%)	
Pension	64/103 (62.1%)	41/66 (62.1%)	
Partnership status			0.729
Marriage	74/103 (71.8%)	44/66 (66.7%)	
Extramarital union	2/103 (1.9%)	2/66 (3%)	
In a relationship	2/103 (1.9%)	1/66 (1.5%)	
Divorced	5/103 (4.9%)	4/66 (6.1%)	
A widower	15/103 (14.6%)	14/66 (21.2%)	
Not in a relationship	5/103 (4.9%)	1/66 (1.5%)	
Feeling their life is endangered due to COVID-19			0.007 * +
Not at all	31/103 (30.1%)	10/66 (15.2%)	
Little	25/103 (24.3%)	9/66 (13.6%)	
Moderately	27/103 (26.2%)	19/66 (28.8%)	
Seriously	15/103 (14.6%)	19/66 (28.8%)	
Very strong	5/103 (4.9%)	9/66 (13.6%)	
Charlson comorbidity index, median and IQR	3 (2–4)	3 (2–4)	0.659
Prevously psychiatrically treated	9/103 (8.7%)	12/66 (18.2%)	0.069 +
Arterial hypertension	61/103 (59.2%)	40/66 (60.6%)	0.858
Diabetes	26/103 (25.2%)	16/66 (24.2%)	0.883
Hyperlipidemia	20/103 (19.4%)	10/66 (15.2%)	0.479
Adipositas	37/103 (35.9%)	24/66 (36.4%)	0.954
Smoking	6/103 (5.8%)	5/66 (7.6%)	0.753
Alcohol use	6/103 (5.8%)	1/66 (1.5%)	0.249
COPD	5/103 (4.9%)	7/66 (10.6%)	0.219
Asthma	4/103 (3.9%)	4/66 (6.1%)	0.713
Heart failure	16/103 (15.5%)	8/66 (12.1%)	0.535
Atrial fibrillation	10/103 (9.7%)	5/66 (7.6%)	0.634
Coronary disease	12/103 (11.7%)	11/66 (16.7%)	0.353
Periferial arterial disease	3/103 (2.9%)	2/66 (3%)	1.000
Cronical renal disease	13/103 (12.6%)	2/66 (3%)	0.032 *
Cronical hepatal disease	2/103 (1.9%)	1/66 (1.5%)	1.000
Active malignant disease	12/103 (11.7%)	5/66 (7.6%)	0.390
Transplanted organ	6/103 (5.8%)	1/66 (1.5%)	0.249
Rheumatic disease	4/103 (3.9%)	5/66 (7.6%)	0.315
WHO severity of COVID-19 at admission			0.678
Mild	10/103 (9.7%)	3/66 (4.5%)	
Moderate	3/103 (2.9%)	2/66 (3%)	
Heavy	80/103 (77.7%)	54/66 (81.8%)	
Critical	10/103 (9.7%)	7/66 (10.6%)	
MEWS symptom intensity, median and IQR	2 (1–4)	3 (2–4)	0.202
Pneumonia	89/103 (86.4%)	63/66 (95.5%)	0.056
Duration of disease symptoms, median and IQR	15 (11–20.5)	15 (11–21)	0.946
ECOG functional status, median and IQR	2 (1–2)	2 (1–3)	0.157
Peak required oxygen flow during hospitalization, median and IQR	6 (3–15)	8 (4–15)	0.218
Duration of hospitalization, median and IQR	12 (8–19)	12.5 (9–18.75)	0.995
Treatment in the intensive care unit	14/103 (13.6%)	11/66 (16.7%)	0.583
Treatment with mechanical ventilation	6/103 (5.8%)	3/66 (4.5%)	1.000
Bacteremia	6/103 (5.8%)	6/66 (9.1%)	0.541
Arterial thrombosis	1/103 (1%)	2/66 (3%)	0.561
Venous thrombosis	10/103 (9.7%)	10/66 (15.2%)	0.285
Heavy bleeding	2/103 (1.9%)	0/66 (0%)	0.521
Death during hospitalization	6/103 (5.8%)	3/66 (4.5%)	1.000
Leukocytes (×10^9^/L), median and IQR	7.5 (5.75–11.9)	8.5 (5.6–10.9)	0.796
Absolute number of neutrophil granulocytes (×10^9^/L), median and IQR	6.3 (4.27–9.87)	7 (4.49–9.72)	0.722
Absolute number of lymphocytes (×10^9^/L), median and IQR	0.9 (0.55–1.38)	0.8 (0.6–1.05)	0.244
Hemoglobin (g/L), median and IQR	134 (114–142.5)	135 (126–147)	0.108
RDW (%), median and IQR	14 (13.3–15.15)	13.6 (13.2–14.3)	0.026 * +
Platelets (×10^9^/L), median and IQR	223 (165.5–299.5)	251 (177–342)	0.139
NLR, median and IQR	8.3 (3.88–13.15)	7.3 (4.59–11.86)	0.673
SII, median and IQR	1709.1 (777.66–3812.13)	1816.9 (969.78–3608)	0.564
CRP (mg/L), median and IQR	68.3 (26.55–127.6)	84 (38.3–164.73)	0.140
IL6 (pg/mL), median and IQR	27 (10.9–84.69)	9.3 (5.36–25.92)	0.151
Ferritin (µg/L), median and IQR	836 (373–1527.5)	605 (373.5–1495.25)	0.754
D-dimers (mg/L FEU), median and IQR	1.1 (0.6–3.37)	0.9 (0.59–1.88)	0.421
Procalcitonin (ng/mL), median and IQR	0.2 (0.08–0.26)	0.1 (0.06–0.2)	0.048 * +
Creatinine (mmol/L), median and IQR	83 (67.25–110.25)	74 (63–85)	0.015 *

* statistically significant at level *p* < 0.05 when the Impact of Events Scale-Revised (IES-R) score is stratified according to the absence or presence of symptoms. + statistically significant at level *p* < 0.05 when IES-R score is used as a continuous variable. Abbreviations: PTSD—post-traumatic stress disorder; IQR—interquartile range; COVID-19—Coronavirus disease 2019; COPD—chronic obstructive pulmonary disease; WHO—the World health organization; MEWS—modified early warning score; ECOG—Eastern cooperative oncology group scale; RDW—red blood cell distribution width; NLR—neutrophil to lymphocyte ratio; SII—systemic inflammatory index derived from NLR multiplied by platelet count; CRP—C reactive protein; IL—interleukin; FEU—fibrin equivalent units.

**Table 5:** Multivariate logistic regression models evaluating independent contribution of COVID-19 severity, age, sex, Charlson comorbidity index, and previous psychiatric treatment to individual psychiatric symptoms.

	Depression	Anxiety	Stress	PTSD
MEWS symptom intensity	aOR 1.66 (1.25–2.21); *p* < 0.001 *	aOR 1.04 (0.84–1.3); *p* = 0.705	aOR 0.92 (0.69–1.21); *p* = 0.545	aOR 1.15 (0.91–1.45); *p* = 0.236
Peak required oxygen flow (L/min)	aOR 0.99 (0.98–1.01); *p* = 0.700	aOR 0.99 (0.98–1.0); *p* = 0.679	aOR 1.01 (0.99–1.03); *p* = 0.064	aOR 0.99 (0.98–1.01); *p* = 0.983
Age (years)	aOR 1.0 (0.96–1.04); *p* = 0.969	aOR 1.01 (0.98–1.05); *p* = 0.480	aOR 0.99 (0.95–1.05); *p* = 0.986	aOR 1.03 (0.98–1.06); *p* = 0.172
Female sex	aOR 1.99 (0.92–4.29); *p* = 0.078	aOR 1.96 (1.0–3.83); *p* = 0.047 *	aOR 3.09 (1.38–6.93); *p* = 0.006 *	aOR 3.59 (1.81–7.15); *p* < 0.001 *
Charlson comorbidity index	aOR 1.04 (0.83–1.29); *p* = 0.743	aOR 1.02 (0.83–1.26); *p* = 0.813	aOR 0.84 (0.62–1.15); *p* = 0.132	aOR 0.88 (0.69–1.12); *p* = 0.311
Prevously psychiatrically treated	aOR 0.8 (0.27–2.34); *p* = 0.685	aOR 1.97 (0.71–5.49); *p* = 0.192	aOR 2.21 (0.78–6.24); *p* = 0.131	aOR 1.98 (0.74–5.28); *p* = 0.168

* statistically significant at level *p* < 0.05. Adjusted odds ratios with respective 95% confidence intervals in brackets together with respective *p*-values are presented. Abbreviations: PTSD—post-traumatic stress disorder; MEWS—modified early warning score; COVID-19—Coronavirus disease 2019; aOR—adjusted odds ratio.

## Data Availability

Data are available per reasonable request from the corresponding author.
